# Increased alcohol‐specific mortality in Germany during COVID‐19: State‐level trends from 2010 to 2020

**DOI:** 10.1111/dar.13573

**Published:** 2022-11-09

**Authors:** Carolin Kilian, Sinclair Carr, Bernd Schulte, Jakob Manthey

**Affiliations:** ^1^ Institute of Clinical Psychology and Psychotherapy Technische Universität Dresden Dresden Germany; ^2^ Institute for Mental Health Policy Research Centre for Addiction and Mental Health Toronto Canada; ^3^ Centre for Interdisciplinary Addiction Research, Department of Psychiatry and Psychotherapy University Medical Centre Hamburg‐Eppendorf Hamburg Germany; ^4^ Department of Psychiatry, Medical Faculty University of Leipzig Leipzig Germany

**Keywords:** alcohol, alcohol‐specific mortality, COVID‐19, Germany, pandemic

## Abstract

**Introduction:**

The COVID‐19 pandemic may have led to an increase in the alcohol‐specific mortality. Against this backdrop, the aim of this report is to explore alcohol‐specific mortality trends in Germany of the years 2010 to 2020.

**Method:**

Alcohol‐specific mortality data aggregated by sex, 5‐year age groups and state were collected from the annual cause‐of‐death statistics and analysed descriptively by visual inspection.

**Results:**

The overall alcohol‐specific mortality rate (age‐standardised) has mainly decreased between 2010 and 2020. However, increased alcohol‐specific mortality rates for the year 2020 compared to 2019 were found for both, women (+4.8%) and men (+5.5%), particularly in age groups between 40 and 69 years. Changes in alcohol‐specific mortality rates differed between federated states, with steeper increases in East Germany.

**Discussion and Conclusions:**

Different mechanisms related to the increase in alcohol consumption, particularly among high‐risk drinkers, and reduced resources in health care may have led to an increase in alcohol‐specific mortality in Germany in 2020. Despite the recent decline in the alcohol‐specific mortality in Germany, an increase in the death toll was observed in 2020.

**Key points**

- Age‐standardised alcohol‐specific mortality rates decreased continuously in Germany between 2016 and 2019.
- The alcohol‐specific mortality rate in Germany increased by 5.3% in 2020 compared to the previous year.
- An increase in the alcohol‐specific mortality rate was observed in both men and women, and was most pronounced among those aged 40 to 69 years.
- State‐level differences were found in the trend reversal of alcohol‐specific mortality rates in 2020, with more pronounced increases in Eastern German states.

## 1 INTRODUCTION

Alcohol use is prevalent in Europe and causally linked to numerous diseases [1]. In 2016, about 265,000 deaths were estimated to be attributable to alcohol use in the European Union (EU), accounting for about 5% of all registered deaths. In the past decade, both alcohol use and alcohol‐specific harm, indicated by alcohol‐attributable deaths, showed declining trends in Europe [2, 3], marking considerable progress towards the sustainable development target 3.5 (indicator 3.5.2).

In Germany, per capita consumption has declined continuously, even if slightly, since 2000. Between 2010 and 2019, sales data indicate a decrease from 11.6 to 10.6 L of pure alcohol [3]. Given the very liberal alcohol control policy framework in Germany, this decline is remarkable and may reflect changes in drinking habits [4]. In line with that, repeated cross‐sectional data suggest a moderate decline in youth drinking between 1999 and 2015 across European sub‐regions including Germany [5] and according to the German Epidemiological Survey of Substance Abuse, alcohol use appears to decline particularly among men, while heavy drinking episodes have increased among women in recent years [6].

In the course of the COVID‐19 pandemic, however, a set of unprecedented restrictions were introduced in Germany in 2020. These included not only the closure of restaurants and bars, as well as the cancellation of many private and public festivities, but also temporary and regional restrictions of alcohol sales. Moreover, public drinking was prohibited in some municipalities during the first and second wave in March/April and November/December 2020. As opposed these restrictive measures, the value‐added tax was lowered in the second half of 2020 to stimulate private consumption, with the consequence that alcoholic beverages became more affordable [7].

The question of how these COVID‐19‐related measures may have impacted alcohol use in Germany and elsewhere has been of remarkable interest to the scientific community since the onset of the pandemic. According to a meta‐analysis compiling the evidence on COVID‐19‐related changes in alcohol use, about the same proportions of individuals reported reductions and increases in consumption since the pandemic's onset [8]. Additionally, substantial declines in heavy episodic drinking were found among more than 30,000 past‐year drinkers surveyed across Europe, resulting in—on average—lower alcohol intake levels in the surveyed population [9]. The observed reductions in alcohol use were less pronounced in some countries including Germany [10]. Moreover, people with very high consumption levels were more likely to report increases than decreases in their alcohol use, raising concerns about increasing rates of alcohol harm [11].

Apart from the impact on alcohol use, the pandemic, and in particular the mitigation measures, had substantial effects on the delivery of routine health care. In Germany, a considerable decline in consultations in primary care and outpatient services was registered in spring and autumn 2020, when severe contact restrictions were in place [12]. In a general population survey, 26–30% of those in need for care reported problems accessing medical care in spring 2020, while about 15% of an independent sample of individuals in need for care reported health deteriorations [13]. Data from Spain show lower service use rates among people with alcohol use disorders [14]. Thus, people in need of treatment for health issues caused by their drinking may have found it more difficult to find appropriate support during the pandemic, increasing the mortality risk in this already vulnerable population.

So far, studies on changes of alcohol‐specific harm during the pandemic are very limited. Recent death statistics for England and Wales for 2020 show a 19.6% increase in alcohol‐specific mortality compared to the previous year, marking the highest number of recorded alcohol‐specific deaths since 2001 [15]. Increased levels of alcohol intake, especially among people with high‐risk drinking levels, is considered to be a driver for the increase in deaths from alcohol‐specific liver diseases [16]. A modelling study supported this association showing that a short‐term increase in alcohol use during the pandemic can significantly increase long‐term morbidity and mortality from alcohol‐specific liver diseases [17].

In light of the increased alcohol use among people with high‐risk drinking levels and limited access to health care in 2020, we expect alcohol‐specific mortality to have increased among both women and men in contrast to trends of previous years. To investigate this hypothesis, we sought to explore sex‐specific trends in alcohol‐specific mortality in Germany between 2010 and 2020. Alcohol‐specific causes of death include several diagnoses, most of which indicate very heavy chronic drinking, such as alcoholic liver disease, alcoholic cardiomyopathy and alcohol poisoning. We further disaggregate sex‐specific trends by age group and federated state to gain additional insights into alcohol‐specific mortality changes.

## 2 METHODS

### 2.1 Data source

We obtained aggregated mortality data from the annual cause‐of‐death statistics, provided by the *Information System of the Federal Health Monitoring* [18]. We selected only alcohol‐specific deaths (diagnoses according to International Classification of Diseases, 10th revision [19]: F10, G31.2, G62.1, G72.1, I42.6, K29.2, K70, K85.2, K86.0, Q86.0, T51.0, T51.9) and extracted two mortality outcomes—death tolls and mortality rates—by sex and either for 5‐year age groups or for federated states for the years 2010 to 2020. The alcohol‐specific conditions chosen have in common that changes in consumption can be reflected in mortality in the short term, that is, within a single year [20]. The state‐specific mortality rates were age‐standardised to the standard population in Germany in 2011 [18].

### 2.2 Data analyses

Due to the insufficient number of data points, time series analysis was not possible. Instead, we focused merely on descriptive analyses to evaluate sex‐specific changes in alcohol‐specific death tolls and mortality rates between 2010 and 2020. Our analyses started with the total number of deaths registered over the past 11 years in order to identify temporal patterns. Next, we studied the presence of any observed patterns by age group and by federated state. As there were only very few alcohol‐specific deaths among 0‐ to 29‐year‐olds (especially among women), we limited the age‐specific analyses to individuals aged 30 years or older.

All analyses were performed in R version 4.0.5 [21]. The data and R script that support the findings of this study are openly available in the Figshare respiratory at https://figshare.com/projects/Trends_in_alcohol‐specific_mortality_in_Germany_2010‐2020/129551.

## 3 RESULTS

### 3.1 Temporal patterns in alcohol‐specific mortality between 2010 and 2020

In 2020, a total of 14,218 alcohol‐specific deaths were registered in Germany. More than 90% of these deaths were due to alcohol use disorders (any F10 diagnosis) or alcoholic liver diseases (any K70 diagnosis).

**FIGURE 1:**
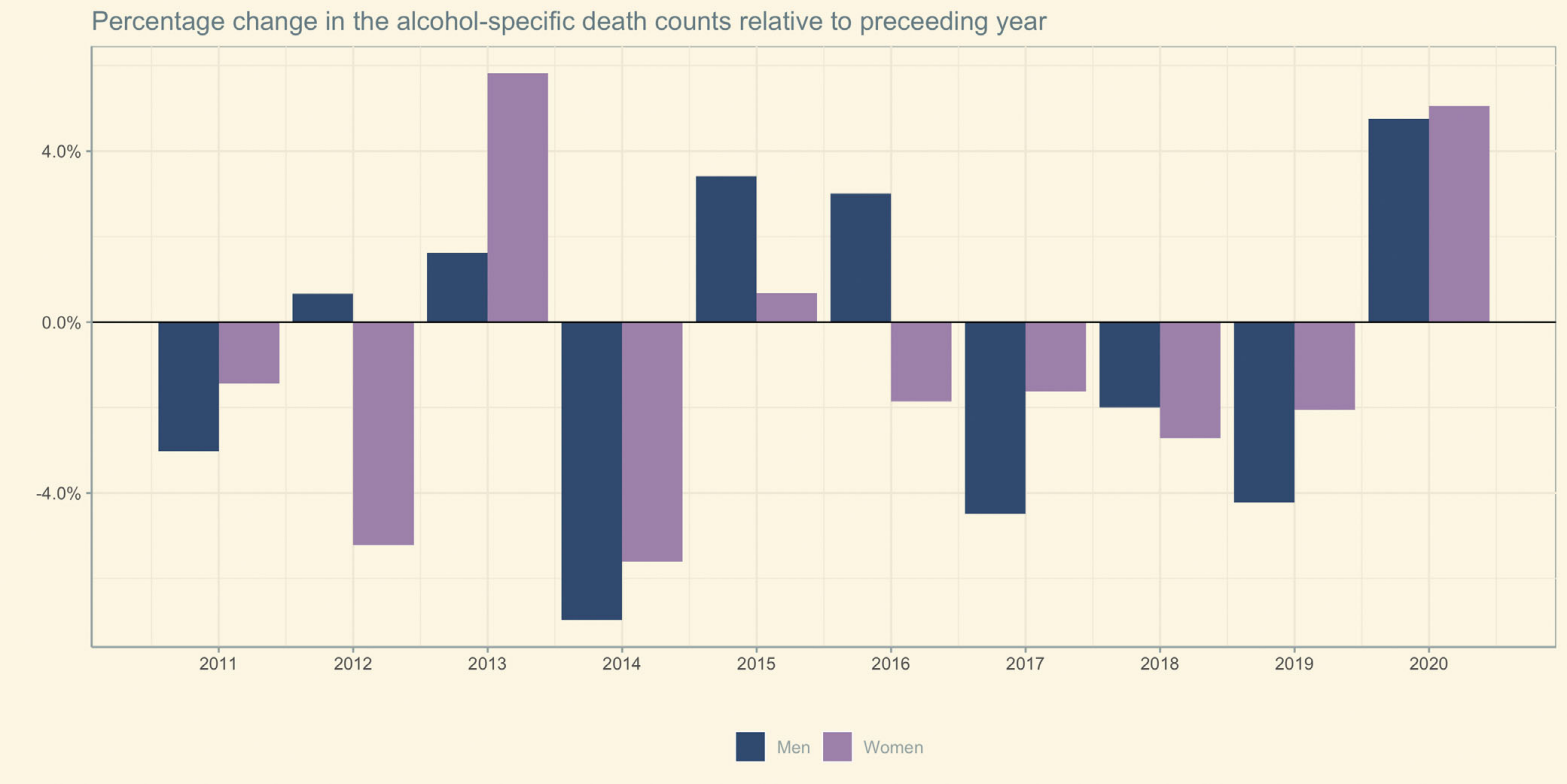
Relative change in the number of alcohol‐specific deaths to the preceding year among women and men in Germany between 2011 and 2020.

Between 2010 and 2020, the overall alcohol‐specific death toll decreased, as summarised in Table 1 and illustrated in Figure 1. Only in 2013 (mainly driven by an increase in female mortality), 2015 and 2016 (in both years almost entirely driven by male mortality), the death toll has increased in comparison to the preceding year. Between 2016 and 2019, the number of deaths continuously decreased.

**TABLE 1:** Alcohol‐specific mortality in Germany between 2010 and 2020

	Total		Women		Men	
Year	Number of deaths	Age‐standardised death rate^a^	Number of deaths	Age‐standardised death rate^a^	Number of deaths	Age‐standardised death rate^a^
2010	15,031	18.4	3863	9.3	11,168	27.9
2011	14,658	18.3	3809	9.3	10,849	27.7
2012	14,551	18.1	3629	8.8	10,922	27.8
2013	14,973	18.6	3868	9.4	11,105	28.1
2014	14,099	17.4	3673	8.9	10,426	26.3
2015	14,506	17.8	3698	8.9	10,808	26.9
2016	14,786	18.8	3632	8.7	11,154	27.5
2017	14,270	17.3	3575	8.5	10,695	26.2
2018	13,973	16.9	3483	8.3	10,490	25.6
2019	13,496	16.2	3414	8.1	10,082	24.6
2020	14,218	17.1	3606	8.6	10,612	25.9

^a^ Per 100,000 persons.

In 2020, 813 fewer deaths were registered compared with 2010 (−5.7%), but 722 more deaths compared with 2019 (+5.3%). The relative increase in alcohol‐specific mortality in 2020 was similar to the increase in all‐cause mortality rates (+4.9%). As illustrated in Figure 1, increasing death tolls in 2020 were about the same in women (+5.5%) and men (+4.8%). Within the last decade, the annual alcohol‐specific mortality for both men and women (except in 2013) never increased at such a rate as in 2020.

### 3.2 Age‐specific trends

**FIGURE 2:**
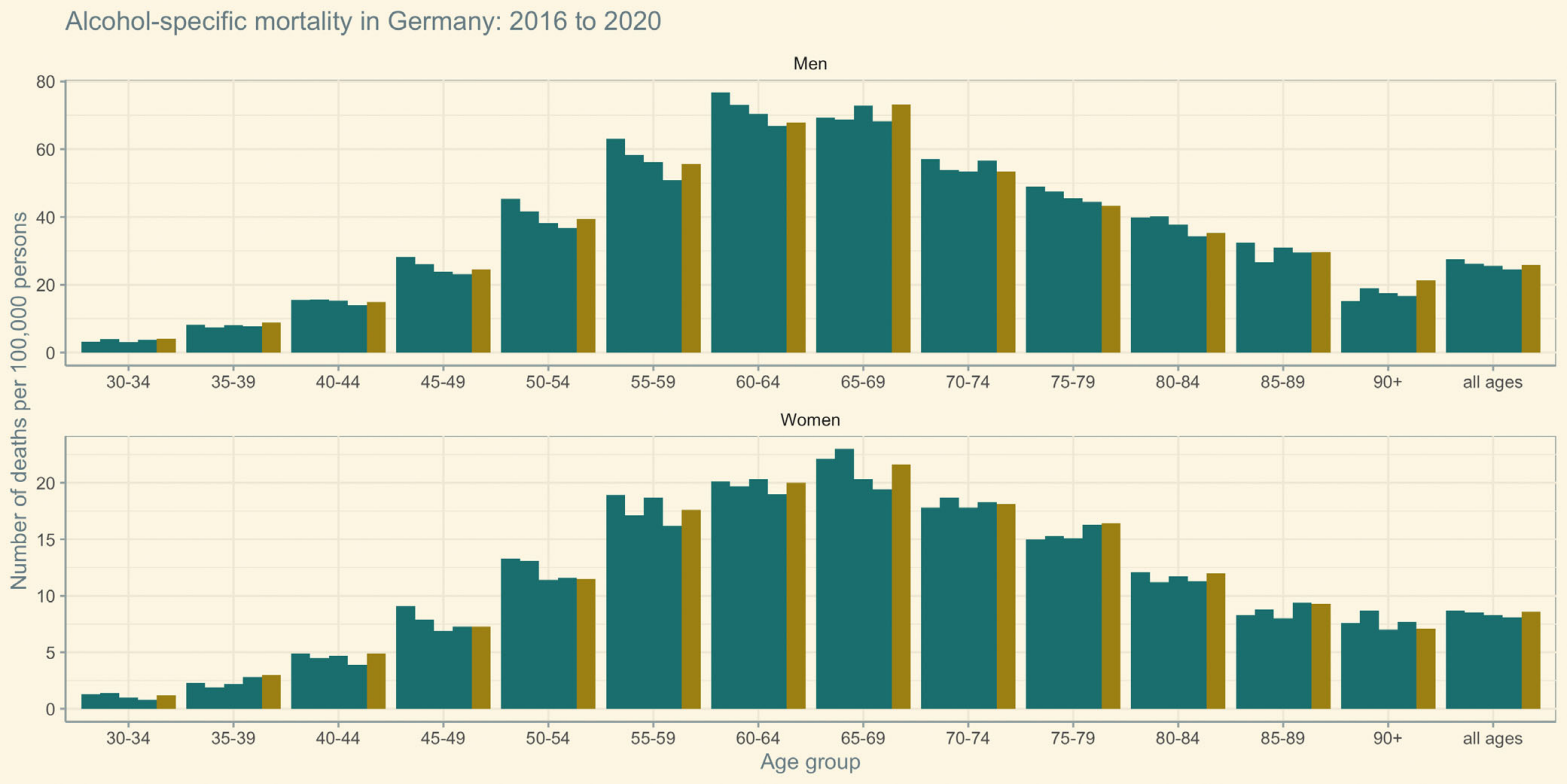
Mortality rates between 2016 and 2020 among German women and men by age group. Green bars indicate the years 2016 to 2019 and the yellow bar indicates the year 2020.

We plotted alcohol‐specific mortality by age groups between 2016 and 2020 to identify age groups which were mainly responsible for the observed increase in alcohol‐specific mortality in 2020. We chose the time period 2016 to 2020 as it represents the most recent trends and was identified as a period with a continuous decline in mortality rates. As illustrated in Figure 2, declining mortality trends between 2016 and 2019 could be observed in most age groups but were discontinued in 2020. Among men, the most pronounced percentage increases in alcohol‐specific mortality rates in 2020 compared to 2019 were present in 45‐ to 69‐year‐olds (+1.5 to 7.8%) and 90+ year‐olds (+4.7%). Among women, the age groups 40 to 44 (+16.9%) and 55 to 69 (+4.7 to 9.2%) registered the largest increases in alcohol mortality rates.

Importantly, the declining mortality trend observed for most age groups between 2016 and 2019 was not present among 35‐ to 44‐year‐olds. In contrast, alcohol‐specific mortality stagnated or even increased (as was for women aged 35 to 39). In 2020, alcohol‐specific deaths (continued) to increase in these age groups.

### 3.3 State‐specific trends

**FIGURE 3:**
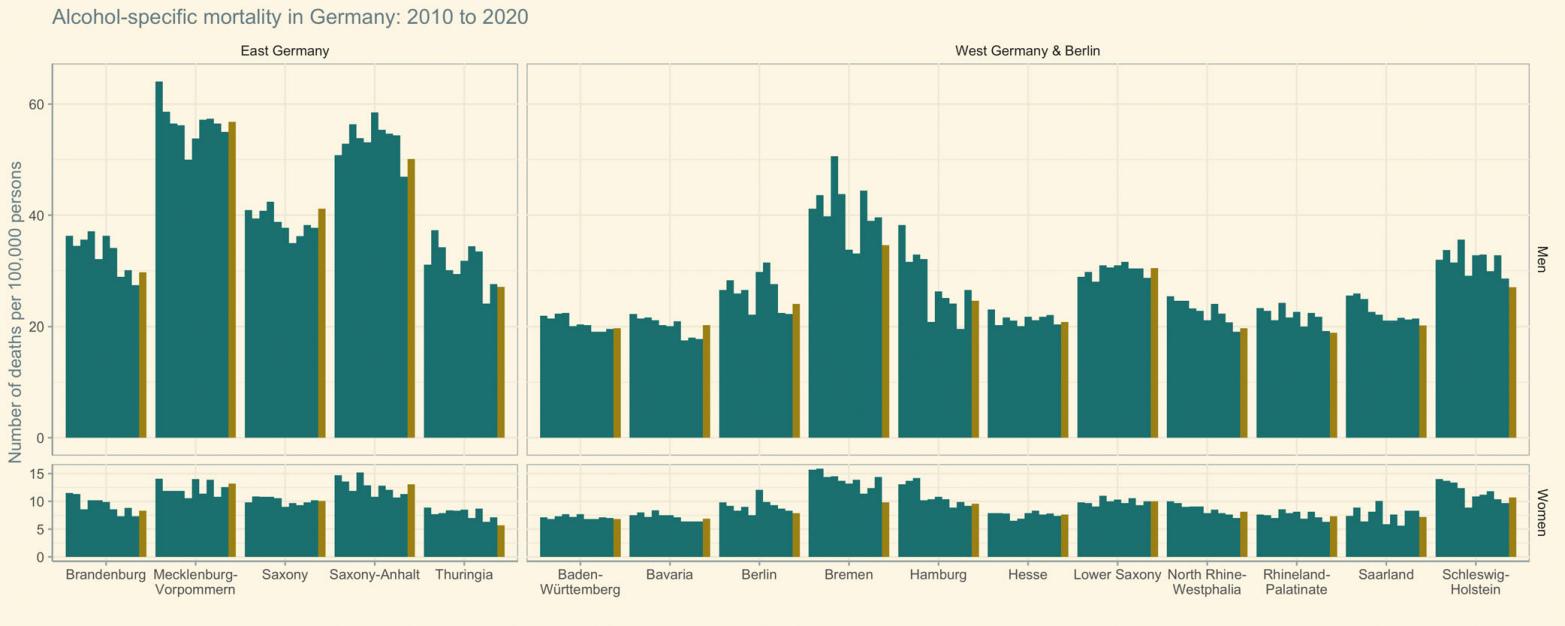
Age‐standardised mortality rates between 2010 and 2020 among women and men by German federated state. Green bars indicate the years 2010 to 2019 and the yellow bar indicates the year 2020.

In order to identify the federated states contributing most to the observed increase in 2020, we plotted alcohol‐specific mortality between 2010 and 2020 by state. We chose this period because the state‐level trends were more heterogeneous than trends observed at the federated state level. The year‐to‐year variations in the state‐level mortality render it difficult to identify unique trends. However, as illustrated in Figure 3, age‐standardised alcohol‐specific mortality rates tended to decline or stagnate in most federated states in the years 2010 to 2019, except for men in Saxony‐Anhalt, and were considerably higher in East Germany (2020: 24.9 deaths per 100,000 persons) as compared to West Germany (including Berlin; 2020: 14.8 deaths per 100,000 persons).

To quantify the magnitude of change in the year of the pandemic, we compared the sex‐ and state‐level alcohol‐specific mortality rates in 2020 with the average of those from 2017 to 2019. We chose this period to account for the variations in the state‐level alcohol‐specific mortality rates in prior years. An increase in the alcohol‐specific mortality rates were observed among men in East Germany (+2.1%), as well as among women in East (+2.9%) and West Germany (+1.7%, including Berlin). Among men in West Germany, a marginal reduction was observed only (−0.5%).

State‐level comparisons revealed a considerable variability in the alcohol‐specific mortality rates across federated states. Among men, the most pronounced increases were observed in Bavaria (+11.1%) and Saxony (+8.5%), while the alcohol‐specific mortality rates were lower in 2020 compared to 2017–2019 in Bremen (−22.7%), Schleswig‐Holstein (−14.6%) and Rhineland‐Palatinate (−13.2%). Among women, higher mortality rates were found in Saxony‐Anhalt (+11.7%), North Rhine‐Westphalia (+6.9%), Bavaria (+6.8%), Brandenburg (+5.7%) and Mecklenburg‐Vorpommern (+5.5%), and lower rates in Bremen (−42.7%), Thuringia (−41.3%) and Berlin (−12.3%).

## 4 DISCUSSION

### 4.1 Principal findings

The aim of this study was to explore trends in the alcohol‐specific mortality in Germany between 2010 and 2020 and, specifically, to investigate a possible increase in mortality in 2020 related to the COVID‐19 pandemic. According to our findings, alcohol‐specific mortality has mostly decreased since 2010 (see also [22]) and more consistently between 2016 and 2019. However, a 5% increase of the death toll attributable to heavy and chronic alcohol use was registered in 2020. This increase was observed for both men and women, and was most pronounced among those aged 40 to 69 years. At the federated state level, both increases and declines in alcohol‐specific mortality rates were identified, with a general increase among men and women in East, as well as among women in West Germany.

### 4.2 Interpretation of our findings

Although more in‐depth analyses of alcohol‐specific mortality data are needed to confirm such a trend, our findings are in line with preliminary data from England and Wales that indicate a 19.6% increase in the alcohol‐specific mortality rate in 2020 compared to 2019 [15]. A similar yet more pronounced trend was observed in the United States, where the alcohol‐specific mortality rate increased by 25.9% ([23], see also [24]). While the increase in the alcohol‐specific mortality in Germany was smaller compared with that observed in England, Wales and the United States, it is plausible that similar drivers are behind the observed upturn. These drivers may include an increase in alcohol use among individuals who already drank alcohol at high‐risk levels before the pandemic [11] and increased rates of alcohol relapses among alcohol‐dependent but previously abstinent people [25]. There is robust evidence that the mortality risk increases with higher amounts and frequencies of alcohol use, particularly among those with heavy episodic drinking [1]. Additionally, recent model projections from the United States showed that a short‐term increase in alcohol consumption due to COVID‐19 could lead to an increase in alcohol‐specific liver‐related morbidity over a period of two decades and additional deaths in subsequent years [17].

In addition to short‐term increases in alcohol use, other factors exist that may have contributed to the trend reversal in alcohol‐specific mortality. With rising numbers of COVID‐19 infections and deaths, the health‐care system was (and still is) heavily burdened, and comprehensive medical care for other conditions was at times not secured [26]. In Germany, the federated states were affected to varying degrees by the COVID‐19 pandemic, with some states, such as Saxony, experiencing a particularly high number of COVID‐19 infections and deaths [27]. Regional differences in COVID‐19 burden, as well as in health‐care provision, may have contributed to the observed differences in alcohol‐specific mortality rates between the federated states.

It is noteworthy that the state‐level results for the eastern German states converge towards a general increase in alcohol‐specific mortality in 2020. Given that alcohol‐specific mortality, as well as all‐cause mortality [28], was already higher in this part of Germany before COVID‐19, a probable increase in mortality in 2020 is alarming. Several reasons for the persistent differences in the mortality between East‐ and West‐Germany have been discussed, including accessibility and quality of medical care particularly in peripheral regions in the East [29, 30], a higher prevalence of lifestyle risk factors such as smoking and alcohol [31], as well as socioeconomic consequences of the reunification due to the higher rates of interrupted employment histories in East Germany [32].

### 4.3 Limitations

There are a number of limitations and caveats to our findings. First, the data in this study are based on causes‐of‐death statistics, which are prone to inaccuracies [33]. Second, we were not able to include measures of uncertainty in our analyses as these are not part of cause‐of‐death statistics. The only key source of variation for alcohol‐specific deaths may be related to inconsistent coding practice [34] but there is no data available to control for this phenomenon. Third, in addition to deaths in which alcohol is the proximate cause, alcohol also contributes to a number of other diseases and external causes of morbidity and mortality, such as road traffic accidents, homicides and suicides, resulting in an under‐representation of the true burden, especially among younger men [35]. Finally, our study was of an exploratory and descriptive nature, as systematic analyses of possible causes of changes in alcohol‐related mortality were beyond the scope of our research. Therefore, we would like to point out that our results merely provide an overview of sex‐ and age‐specific, as well as state‐level trends in alcohol‐specific mortality in Germany between 2010 and 2020 and do not allow any inferences on possible causes.

## 5 CONCLUSION

In Germany, alcohol‐specific mortality increased in 2020 compared to the preceding year, indicating a possible trend reversal in decreasing alcohol‐specific mortality rates since 2016. This increase varied by age groups and federated states, with more pronounced increases among middle‐aged adults and in Eastern German states. Given the ongoing pandemic situation and a much higher COVID‐19 burden in Germany in 2021 compared to the first pandemic year, we expect further increases in the alcohol‐specific mortality rates in the following year.

## AUTHOR CONTRIBUTIONS

Conceptualisation: Carolin Kilian, Jakob Manthey; Methodology: Carolin Kilian, Jakob Manthey; Formal analysis and investigation: Carolin Kilian, Jakob Manthey, Sinclair Carr; Writing—original draft preparation: Carolin Kilian, Jakob Manthey, Bernd Schulte; Writing—review and editing: all authors; Supervision: Jakob Manthey.

## CONFLICT OF INTEREST

None to declare.

## ETHICS STATEMENT

In this study, publicly available data was analysed, which can be accessed through the sources indicated.
